# Boiling, Blanching, and Stir-Frying Markedly Reduce Pesticide Residues in Vegetables

**DOI:** 10.3390/foods11101463

**Published:** 2022-05-18

**Authors:** Kamonrat Phopin, Sompon Wanwimolruk, Chosita Norkaew, Jaruwat Buddhaprom, Chartchalerm Isarankura-Na-Ayudhya

**Affiliations:** 1 Center for Research and Innovation, Faculty of Medical Technology, Mahidol University, Bangkok 10700, Thailand; kamonrat.php@mahidol.ac.th (K.P.); chosita.nor@student.mahidol.ac.th (C.N.); jaruwat.bud@mahidol.ac.th (J.B.); 2 Department of Clinical Microbiology and Applied Technology, Faculty of Medical Technology, Mahidol University, Bangkok 10700, Thailand; chartchalerm.isa@mahidol.ac.th

**Keywords:** pesticide residues, food safety, cooking, Chinese kale, yard long beans

## Abstract

Nowadays, a lot of produce (fruits and vegetables) sold in many countries are contaminated with pesticide residues, which cause severe effects on consumer health, such as cancer and neurological disorders. Therefore, this study aims to determine whether cooking processes can reduce the pesticide residues in commonly consumed vegetables (Chinese kale and yard long beans) in Thailand. For cooking experiments, the two vegetables were cooked using three different processes: boiling, blanching, and stir-frying. After the treatments, all cooked and control samples were subjected to extraction and GC-MS/MS analysis for 88 pesticides. The results demonstrated that pesticide residues were reduced by 18–71% after boiling, 36–100% after blanching, and 25–60% after stir-frying for Chinese kale. For yard long beans, pesticide residues were reduced by 38–100% after boiling, 27–28% after blanching, and 35–63% after stir-frying. Therefore, cooking vegetables are proven to protect consumers from ingesting pesticide residues.

## 1. Introduction

Pesticides are widely employed in agriculture and in many developing countries, and most farmers do not utilize pesticide products in accordance with the Good Agricultural Practices (GAP). GAP are a set of standards for the safe and sustainable production of crops and livestock, which aims to help farmers maximize yields and optimize business operations while minimizing production costs and environmental impact. Upon application of pesticide, pesticide residues remain on fruits and vegetables and are toxic. The ingestion of fruits and vegetables contaminated with pesticide residues is one of the main ways humans are exposed [1]. Exposure to pesticide residues has been attributed to an increased risk of developing cancer and the dysfunction of reproductive systems in humans [2,3,4,5,6]. There has been growing public awareness and concern regarding pesticide residues in fruits and vegetables and food safety [1,7]. For these reasons and to protect consumers from the harmful effects of pesticides, many developed countries have established strict guidelines including the ‘maximum residue limits’ (MRL) and GAP. These are to regulate the use of pesticides in agriculture. Additionally, routine monitoring and surveillancing of the pesticide residues in food are effectively performed. Some developed countries make food surveys for diets, namely total diet studies, which measure the residue levels after cooking. Therefore, few pesticide residues are typically detected in produce from developed countries, and this is reflected by the lack of harmful effects found in their consumers [8,9,10]. Monitoring and quantification of pesticide residues in food samples are vital to prove that these pesticide residues are lower than the MRL. On the other hand, in developing countries, such as Thailand, GAP are not fully implemented, nor has a program of pesticide monitoring been effectively executed either. Similar to many other countries, pesticides have been used extensively in agriculture in Thailand [3]. As a result, food safety associated with pesticide contamination in fruits and vegetables is a public concern in Thailand [11,12].

Chinese kale (*Brassica alboglabra*, BRASSICACEAE) is a green leafy vegetable that is commonly consumed by Asian people including Thai people. Chinese kale has a flavor similar to that of broccoli, however, somewhat more bitter. This vegetable is commonly used in Chinese cuisine, and in particular, Cantonese cuisine. Many popular Thai dishes have Chinese kale as a principal ingredient and in some dishes, Chinese kale is consumed fresh without cooking. It is potentially toxic if the vegetable is eaten this way, especially daily. Previous studies have shown that pesticides were found in 85% of the Chinese kale sold in Thailand with a 29% rate of pesticide detection above the MRL [11,13,14].

Long bean or yard long bean (*Vigna sesquipedalis* Koern, LEGUMINOSAE) is one of the most widely consumed vegetables in Thailand. Yard long bean is an important crop produced in more than 90 countries around the world [15]. In Thailand, yard long bean is an everyday vegetable, consumed both in fresh (raw) and cooked dishes. Bean is an important source of vitamin K, vitamin C and dietary fiber. Pesticide contamination in yard long beans is rather common as pesticide residues were often found in the yard long bean samples sold in Thailand [16]. The rate of pesticide detection in the yard long bean was 100% [16]. These long bean samples also contained pesticides at levels exceeding the recommended MRL by a rate of 95% [16].

Consumption of fruits and vegetables has been shown to have great benefits in preventing non-communicable diseases (NCD), such as cancer and cardiovascular diseases [5,6,17]. However, intake of fruits and vegetables highly contaminated with pesticide residues has been found to possess less benefit in the prevention of NCD [17,18]. Sandoval-Insausti et al. [17] observed that the consumption of fruits and vegetables with low-pesticide-residue content was associated with a lower risk of total mortality and cause-specific mortality. Hence, the content of pesticide residues in fruits and vegetables appears to be a vital factor in discerning the benefits of fruits and vegetables in preventing NCD. To achieve lower pesticide residue content, and thus increase the benefits from fruits and vegetables, simple household processing, such as washing, peeling and cooking, should be employed. Washing is the simplest method; however, it has been shown to be less effective than cooking vegetables. This is because washing with water only reduced pesticide residues loosely attached to the surface of vegetables [19,20]. Most vegetables, excluding salad vegetables, are cooked prior to consumption. Cooking methods have an advantage in terms of processing with heat at high temperatures. It has been demonstrated that in most cases, cooking resulted in large reductions in the levels of pesticide residues in cooked vegetables [21,22,23,24,25,26,27,28]. Thus, cooking processes have a very important effect on diminishing the pesticide residues in cooked vegetables. Data from previous studies were focused on determining the pesticide residues in fresh vegetables and mainly in Western vegetables [29,30,31]. Less research has been conducted on cooked vegetables and vegetables commonly consumed by Asian people. The purpose of this study was to determine whether cooking reduces pesticide residues in Chinese kale and yard long beans, and consequently, it will also help to minimize the risks associated with ingestion of pesticide residues remaining in these vegetables. The outcome of this study will provide knowledge on how to decrease the risks related to pesticide residues in contaminated vegetables in a domestic setting, specifically in developing countries.

## 2. Materials and Methods

### 2.1. Chemicals and Standards

Standards of eighty-eight pesticides and two metabolites, as listed in Supplementary Materials, were purchased from Dr. Ehrenstorfer (Augsburg, Germany). A different stock of standard solutions (1000 mg/L) was prepared in acetonitrile. The standard solutions were kept frozen at −20 °C until required. HPLC-grade acetonitrile was purchased from Merck (Darmstadt, Germany). Sodium chloride and anhydrous magnesium sulfate were obtained from Ajax Finechem, Australia. PSA (Primary secondary amine) of particle size 40 μm and GCB (graphite carbon black) were purchased from Supelco (Sigma-Aldrich Corp., St. Louis, MO, USA).

### 2.2. Vegetable Samples Cooking Processes

For Chinese kale, the samples were collected from a local vegetable farm in Nakhon Pathom province, Thailand. These samples were harvested two days after the Chinese kale had been sprayed with a mixture formulation of eight pesticides. The formulation consisted of eight pesticides dispensed using 10 L of tap water. These pesticides were fenobucarb (five teaspoons), indoxacarb (one teaspoon), chlorpyrifos (two teaspoons), diazinon (four teaspoons), profenofos (four teaspoons), cypermethrin (five teaspoons), deltamethrin (two teaspoons), and metalaxyl (2.5 teaspoons). The doses of pesticides used were in accordance with those recommended by the Good Agricultural Practices (GAP). The reason behind harvesting the vegetables two days after the pesticides’ application was to mimic the common behavior of most Thai farmers who do not obey the GAP rules. After the Chinese kale samples were delivered to the laboratory, they were randomly divided into four experimental groups. These included controls—uncooked (*n* = 6) Chinese kale, boiled Chinese kale (*n* = 8), blanched Chinese kale (*n* = 6), and stir-fried Chinese kale (*n* = 8). The size of each sample was 150–200 g. Of the 88 pesticides investigated, 16 different pesticides were found in the Chinese kale samples. Eight of them were intentionally sprayed on the vegetable before harvesting. These were fenobucarb (2510 ppb), indoxacarb (105 ppb), chlorpyrifos (1342 ppb), diazinon (1892 ppb), profenofos (5175 ppb), cypermethrin (198 ppb), deltamethrin (318 ppb), and metalaxyl (1179 ppb). Their mean concentrations (*n* = 8) found in the Chinese kale samples are indicated in the parentheses. In addition, there were another eight pesticides detected in these samples. These were carbofuran (8 ppb), fenvalerate (36 ppb), flumethrin (168 ppb), λ-cyhalothrin (78 ppb), flutolanil (0.5 ppb), mepronil (322 ppb), alachlor (8 ppb), and mefenacet (0.7 ppb).

For the yard long beans, the samples were purchased from a local market in Nakhon Pathom province, Thailand. A lot of yard long beans were known to come from the same farm. It was not possible to obtain the yard long bean samples directly from the farm and get them sprayed with the desired pesticide formulation, so an initial test was necessary to ensure that the presence of pesticide residues was adequate. For this, the GC-MS/MS analysis was conducted on the yard long bean samples bought from the local market. If the results showed considerable concentrations of the pesticide residues, the long bean samples would then be subjected to the cooking experiments (*n* = 8 each). The experimental design was similar to that carried out for the Chinese kale samples. The size of each yard long bean sample was 150–200 g.

### 2.3. Cooking Processes

The Chinese kale samples were sliced into small pieces, approximately 1–1.5 inches, and then cooked as follows: for the boiling experiments, 700 mL of distilled water in a stainless-steel pot was boiled and all of the Chinese kale samples (150 g) were added to the boiling water (approximately 98–100 °C). After 10 min, the Chinese kale samples were then taken out and left to cool down to room temperature. In the blanching experiments, the Chinese kale samples were blanched in boiling water (approximately 98–100 °C) for 2 min. The samples were then removed and left to cool down to room temperature. For the stir-frying experiments, vegetable oil was added to a hot frying pan. When the oil reached its boiling point (approximately 220 °C), the Chinese kale samples were added and stir-fried in the pan for 3 min. The vegetable was then removed from the pan and left to cool down to room temperature. The cooking experiments for the yard long bean samples were similar to those described for the Chinese kale samples. One exception was that the yard long bean samples were boiled for 5 min instead of 10 min. The selection of cooking times was based on Thai and Chinese cooking practices, which usually employ shorter cooking times to minimize over-cooking. After the end of each experiment, the excess water and oil on the two vegetables were eliminated using paper towels. These cooked vegetable samples were then subjected to analysis to determine the pesticide residue concentrations. The cooked vegetables were chopped into smaller pieces and then blended using a high-speed food processor and mixed carefully. The homogenized samples were then extracted and analyzed as stated below.

### 2.4. Sample Preparation

Pesticide residues were initially extracted by using the pesticide QuEChERS (Quick Easy Cheap Effective Rugged and Safe) method as portrayed previously [32,33,34,35]. In short, the extraction of pesticides was accomplished by extracting 15 g of homogenized vegetables (Chinese kale or yard long beans) with 15 mL acetonitrile saturated with 6 g of magnesium sulfate and 1.5 g of sodium chloride. This process was then followed by a cleaning up process. For Chinese kale and yard long bean samples, this was conducted by relocating the supernatant (1 mL) into another tube comprising 150 mg magnesium sulfate, 50 mg of primary-secondary amine (PSA) and 7.5 mg graphited carbon black (GCB). After shaking and centrifugation, the extracted supernatant was then transferred to an autosampler vial for direct injection into the Bruker GC-MS/MS system.

### 2.5. GC-MS/MS Analysis

Detection of pesticides was achieved using a Bruker 456 gas chromatography (GC) linked with a Bruker Scion Triple Quadrupole mass spectrometer (GC-MS/MS). Details of the GC-MS/MS settings were as described previously [36]. The method of validation regarding reproducibility, recovery, calibration linear range, the limit of detection (LOD), and limit of quantification (LOQ) was performed for the vegetable matrix as explained earlier [37,38].

### 2.6. Calibration and Quantification

Calibration curves were constructed for each pesticide of interest, using the procedures described previously [34,38]. These were accomplished using the same process each time when a new unknown sample set was assayed. Selected ions of known m/z, which are the most plentiful ions of each pesticide, were used for quantification. The remaining ions were used for confirmation of the analyte identity. The peak area ratio of the pesticide standard to that of the internal standard (triphenyl phosphate, TPP) was used for quantitative assessment. Blank samples of either Chinese kale or yard long beans were employed for recovery studies and for preparation of the matrix-matched multi-level calibration standards. Prior to use, these blank samples were analyzed and recognized to be free of the pesticides of interest.

Pesticide quantitation in unknown vegetable samples was accomplished in duplicate unless specified. In each sample lot, a quality control sample at a concentration of 50 ppb for the matrix was assayed. This is to verify the trustworthiness of the method with respect to the detection of the targeted analytes and the accuracy of the analytical assay. Values of MRL for each pesticide in the Chinese kale and yard long beans were adopted from recommended MRL values inaugurated by the European Commission Pesticide Database (2016) and Central Laboratory Thailand (2016) [39,40].

### 2.7. Statistical Analysis

Outcomes are presented as mean ± standard deviation (SD). Analysis of the data for the differences in parameters among three sample groups (or more) was accomplished by one-way analysis of variance (ANOVA) followed by Tukey’s test. For two sample groups, these were evaluated by either the unpaired Student’s t-test or the Mann–Whitney U-test, dependent on their normality of distribution. *p* values of <0.05 were referredas statistically significant. SPSS package version 18.0 (SPSS Inc., Chicago, IL, USA) was utilized for these statistical analyses.

## 3. Results

### 3.1. GC-MS/MS Method Validation

The 88 pesticides investigated were selected based on their common use in the agriculture of fruits and vegetables in Thailand. The extraction method—QuEChERS followed by GC-MS/MS analysis used in this study, provided satisfactory separation with high sensitivity and good selectivity for quantitative measurement of the 88 pesticides of interest. There were no interfering peaks co-eluted with analytes of interest, observed in the GC-MS/MS chromatograms of blank extracts from both Chinese kale and yard long bean samples (results are not shown). Moreover, in all Chinese kale and yard long bean samples tested, there were no detectable peaks identified with the same retention time as the internal standard, triphenyl phosphate (TPP) employed in our GC-MS/MS analysis. This attests that using TPP as the internal standard for our assay was pertinent. Results of recovery studies and calibration curves of the pesticides seen in both Chinese kale and yard long beans were shown to be satisfactory. The recovery of pesticide residues ranged from 75 to 110%. The calibration curves were shown to be linear with the coefficient of determination (r^2^) values greater than 0.99. Additionally, relative standard deviations (RSD) of less than 20% were observed. The limit of detection (LOD) was found to be 0.001 ppb which was less than the MRL of pesticides. Thus, the extraction and GC-MS/MS methods employed in the current study are applicable for the analysis of pesticide residues in both Chinese kale and yard long bean samples.

### 3.2. Effects of Cooking on Pesticide Residue Removal in Chinese Kale Samples

Table 1 shows the effects of boiling on the removal of pesticide residues in the Chinese kale samples. The pesticide residues were classified according to their chemical classes, carbamates, organophosphates, pyrethroids and others. The boiling of the Chinese kale for 10 min had a considerable effect on the removal of the three carbamates tested. This process removed indoxacarb (71%) better than carbofuran (35%) and had the least effect on fenobucarb (21%). After boiling the Chinese kale for 10 min, two of the organophosphates, diazinon (32%) and profenofos (18%) were removed to a moderate extent (Table 1). Boiling had no effect on the removal of chlorpyrifos (0%) from the Chinese kale. Boiling caused similar moderate effects of removal of the four pyrethroid pesticides, namely λ-cyhalothrin (55%), cypermethrin (56%), deltamethrin (66%) and fenvalerate (62%). In contrast, it had little effect on the removal of the pyrethroid flumethrin (9%) from the Chinese kale.

Blanching the Chinese kale for 2 min caused a moderate removal of three carbamates; carbofuran, fenobucarb and indoxacarb were removed by 69%, 47% and 22%, respectively (Table 2). Blanching had less effect on removing the organophosphate pesticides, as it eliminated only 20% of chlorpyrifos residues from the Chinese kale. Diazinon and profenofos were not removed from the Chinese kale by blanching (Table 2). For pyrethroid pesticides, blanching the Chinese kale for 2 min had moderate removal effects. Pesticide removal by blanching ranged from 37% for λ-cyhalothrin to 45% for deltamethrin. The effect of blanching for removal of other pesticides varied from 100% for alachlor, and 79% for metalaxyl to no effect for flutolanil, mefenacet and mepronil.

The effects of stir-frying on the removal of pesticide residues are presented in Table 3. For the carbamate pesticides, stir-frying caused moderate removal of these three pesticides. The stir-frying process removed 25% of carbofuran residues, 26% of fenobucarb residues, and 35% of indoxacarb residues from the Chinese kale. Stir-frying also had a moderate effect on the removal of the organophosphate pesticides as it removed 31% of chlorpyrifos residues, 42% of diazinon residues, and 40% of profenofos residues. For the pyrethroid pesticides in the Chinese kale samples, stir-frying produced moderate removal of these pesticides ranging from 0% for flumethrin to 60% for λ-cyhalothrin (Table 3). The effect of stir-frying on the removal of other pesticides from the Chinese kale samples, varied from 11% for mepronil to 51% for alachlor and flutolanil.

### 3.3. Effects of Cooking on Pesticide Residue Removal in Yard Long Bean Samples

Five pesticide residues were found in the yard long bean samples used in this experiment. They were three organophosphates (chlorpyrifos, dimethoate and profenofos), one organochlorine (captan), and one pyrethroid (flumethrin). The effects of boiling, blanching and stir-frying on the removal of these pesticide residues from the yard long bean samples are shown in Table 4. After 5 min of boiling, residues of three organophosphates, chlorpyrifos, dimethoate and profenofos were eliminated by 37%, 24%, and 6%, respectively. The organochlorine, captan was effectively removed by 100%, while the pyrethroid (flumethrin) pesticide residues were eliminated by 64% after 5 min of boiling of the yard long bean samples. Two minutes of blanching the yard long bean samples also caused a mild reduction of pesticide residues (Table 4). Organophosphates, chlorpyrifos, dimethoate and profenofos were removed by 27%, 20%, and −7%, respectively, as a result of blanching the yard long bean samples. In contrast, blanching produced considerable removal of the organochlorine, captan (80%) and pyrethroid flumethrin (59%) as illustrated in Table 4. The effects of stir-frying on the removal of pesticide residues from the yard long bean are also shown in Table 4. Mild effects of stir-frying were observed regarding the removal of organophosphate pesticides. Stir-frying removed organophosphate pesticides including chlorpyrifos, dimethoate and profenofos by 19%, −17%, and 6%, respectively. For other pesticides, 10 min of stir-frying eliminated captan by 63% and flumethrin by 34%.

## 4. Discussion

This study employed GC-MS/MS methods to determine the pesticide residues in Chinese kale and yard long beans. These methods were based on the processes previously used for Chinese kale [14]. These involved the QuEChERS sample extraction followed by GC-MS/MS analysis [32,33,34]. These methods were demonstrated to be appropriate and applicable for quantitative analysis of pesticide residues in two commonly consumed vegetables, Chinese kale and yard long bean. These were supported by satisfactory results of assay validation including good sensitivity, selectivity, linear calibration curves, good reproducibility and recovery, and accuracy.

Besides the eight pesticides intentionally sprayed on the Chinese kale samples, there were another eight pesticides found in these samples. These included carbofuran, fenvalerate, flumethrin, λ-cyhalothrin, flutolanil, mepronil, alachlor, and mefenacet. These pesticide residues were commonly detected in Chinese kale sold in Thai markets [14]. Five pesticide residues (chlorpyrifos, dimethoate, profenofos, captan, and flumethrin) were found in the yard long bean samples used in this study. This is consistent with previous findings reported in the yard long bean samples collected from markets in Thailand [14]. Their findings suggested that the yard long bean is one of the vegetables that is commonly highly contaminated with pesticide residues. A variety of pesticide residues found in Chinese kale and yard long bean are not unexpected since this phenomenon has also been seen in other commonly consumed vegetables (e.g., pakchoi, morning glory, cabbage, tomato, and chilli) bought in Thailand markets [12,14].

With respect to the common methods to diminish pesticide contamination in vegetables, washing the vegetables is possibly the easiest and most cost-effective method to achieve this. Many studies have demonstrated that washing the vegetables under running water appreciably removed pesticide residues. However, it should be noted that washing vegetables with water can remove the pesticide residues but not entirely. Earlier studies have shown that washing can diminish pesticide residues loosely attached to the surface of vegetables [19,20]. For instance, by washing tomatoes with water, chlorpyrifos residues were reduced by 41 to 44% [41]. Washing cabbage under running water significantly removed carbofuran residues by 93%, and dimethoate residues by 65% [42]. For other pesticide residues, such as λ-cyhalothrin, fenobucarb and isoprocarb, the amount of removal of these pesticide residues was less than those observed with carbofuran and dimethoate. Washing asparagus with water diminished chlorpyrifos residues in asparagus by 24% [22]. The profenofos residues in the Chinese kale were eliminated by 55% after washing under running water [14]. This suggests that washing with water does not remove all pesticide residues from vegetables. The remaining pesticide residues found after washing were those pesticides that had already been absorbed into plant components and could not be washed away.

For food safety, several processing treatments, such as blanching, boiling, stir-frying, and steaming, etc., have been discovered to be useful ways to degrade numerous pesticide residues [25]. These were subject to the type of pesticide and length of treatment [43]. Upon boiling vegetables of the Brassica rapa type in water for 10 min, dimethoate residues were decreased by approximately 50% [44]. Ordinary washing removed 20–52% of mancozeb residues whereas washing combined with cooking resulted in 53–79% pesticide removal in cabbage, knol khol, tomato, okra and brinjal [45]. Profenofos residues were removed by approximately 100% from aubergines and peppers after blanching and frying [46]. Nath G et al. found that after 10 min of cooking okra, approximately 80% of malathion was removed [47]. Boiling Chinese cabbage for 30 min resulted in the decomposition of diazinon, dieldrin, dimethoate, fenitrothion and chlorothalonil ranging from 72% to 99% [48]. Nagayama reported that residues of organophosphorus pesticides in green tea were reduced during the cooking process [49]. Pesticide residues from contaminated potato tubers were further removed by blanching and frying [50].

The present study has also demonstrated that all three cooking processes, boiling, blanching and stir-frying produced considerable effects on removing the pesticide residues from the two commonly consumed vegetables, Chinese kale and yard long bean. After 10 min boiling of the Chinese kale samples, residues of three pesticide groups, carbamates, organophosphates, and pyrethroids were reduced significantly (18–71%) compared to the uncooked samples (Table 1). Exceptions were chlorpyrifos and flumethrin in which their residues were not significantly removed by boiling. This may be due to higher lipophilicity reflected by Log *p* values of chlorpyrifos and flumethrin when compared with pesticides in the same chemical class (Table 5). These findings aligned with the results obtained from previous studies. Most of these results implied that boiling was effective in diminishing the water-soluble pesticide residues, i.e., with low lipophilicity or low Log *p* values [43]. The relationship between pesticide removal effectiveness and hydrophobicity/lipophilicity is of interest. If the relationship is true, as seen in the case of the removal of chlorpyrifos and flumethrin by boiling in Chinese Kale, it would suggest that low lipophilicity pesticides should be used as they can be easily removed by washing and boiling. Low lipophilicity pesticides could reduce the pesticide residues in vegetables, and their subsequent consumption in everyday diet. Further studies are warranted to validate this idea. Our results with the carbamates group showed inconsistent observations as there was no relationship (r = 0.886; *p* > 0.1) between their ability to remove pesticide residues and Log *p* values (Table 1 and Table 5). Blanching had similar effects as boiling on the removal of pesticide residues from Chinese kale. It had moderate effects on the removal of residues of carbamates and pyrethroids from the vegetable. Two-minute blanching of Chinese kale produced little effect on the removal of organophosphate pesticides whereas blanching markedly reduced the residues of alachlor and metalaxyl. The effects of blanching on the removal of these pesticides appeared to be not related to their lipophilicity as there was no significant relationship (r = −0.475; *p* > 0.2) between the % removal and Log *p* of the pesticides.

Stir-frying of Chinese kale for 3 min provided moderate effects on the removal of carbamate, organophosphate, and pyrethroid pesticide residues. One of the pyrethroid pesticides, flumethrine was not removed by stir-frying. The differences may be related to their lipophilicity diversities since flumethrine is highly lipid soluble with a high Log *p* value (Table 5).

Although the number of pesticide residues evaluated in the yard long bean was less than those studied in the Chinese kale, the results regarding the effects of the cooking processes obtained were similar between the two vegetables. For the yard long bean, boiling caused moderate removal of organophosphate (chlorpyrifos, dimethoate, and profenofos), and pyrethroid pesticides (flumethrin). However, boiling entirely removed an organochlorine pesticide (captan) from the yard long bean samples. Blanching produced moderate to strong effects on the removal of organophosphates (chlorpyrifos and dimethoate), organochlorine (captan), and pyrethroid pesticides (flumethrin). Among the organophosphate pesticides, profenofos was not removed by blanching. This was unlikely to be due to the differences in lipophilicity (Table 5).

Similarly, stir-frying the yard long bean caused mild to moderate effects on the removal of organophosphates (chlorpyrifos and profenofos), an organochlorine (captan), and a pyrethroid pesticide (flumethrin). Another different result observed was that the organophosphate dimethoate was not removed by the stir-frying process. It is suspected that this may be related to the lipophilicity of the chemical pesticides. Both organophosphates, chlorpyrifos and profenofos are relatively lipid soluble with Log *p* values greater than dimethoate (Table 5), thus they are more easily eliminated by heated oil in the stir-frying process. In addition, regarding the selection of the yard long bean, the samples were actually bought from the markets. Unlike the Chinese kale samples, the yard long bean samples were directly used and did not undergo the spraying method. For this reason, the large variations (large uncertainties) in pesticide residues were found in the yard long bean samples. This is attributed to the variation in the results and statistical outcomes (Table 4). Type II errors due to small sizes may also make additional errors in statistical assessment.

In addition, some thermal processing including boiling and stir-frying can concentrate pesticide residues or convert the residues into more toxic metabolites in food [29], in particular, using Chinese traditional cooking. In their study, the effect of Chinese traditional cooking (washing, blanching, stir-frying, frying and combined operations) on eight pesticides residues (pyridaben, procymidone, chlorothalonil, difenoconazole, α-cypermethrin, bifenthrin, S-fenvalerate and λ-cyhalothrin) was investigated in cowpea which was one of the most important bean crops in China. The results have shown that washing and blanching could reduce residues with low Kow while stir-frying and frying were more effective than residues with high Kow. They concluded that blanching (5 min) followed by stir-frying (3 min) was the most effective combined operation in terms of food safety [29]. The Kow is referred to as the partition coefficient of a pesticide in oil and water. Thus, Kow is similar to Log *p* which both reflect the lipid solubility of the pesticide. Our results are consistent with those reported by showing that stir-frying was effective to remove pesticide residues with high lipid solubility [29].

Regarding food safety, the MRL and GAP regulations have been properly implemented in developed countries. These warrant the appropriate use of pesticides and the safety of customers. In contrast, in developing countries including Thailand, the national monitoring program of pesticide residues is not customarily implemented [11,14]. Consequently, there are eminent incidences of pesticide detection in vegetables sold in Thailand; it is vital to assure adequate information is provided to consumers. This important matter is an issue for the Thai government authorities including the Thai FDA and the Department of Agriculture. Thus, it is crucial to deliver knowledge on food safety, especially how to reduce the ingestion of pesticide residues and their risks. Washing and cooking processes are common methods to reduce the risk encountered by consumers. The outcomes of the present and previous studies have proven that ordinary cooking processes including boiling, blanching and stir-frying are effective ways to remove pesticide residues. This knowledge will help protect consumers from ingesting harmful pesticide residues in foods, such as vegetables. Furthermore, the safety practice of washing and cooking can provide confidence to people who are afraid of eating pesticide contaminated fruits and vegetables and help to change their attitude and return to eating more fruit and vegetables.

## 5. Conclusions

In summary, this study confirms that cooking practices, namely blanching, boiling and stir-frying help to reduce pesticide residues in two commonly consumed vegetables, Chinese kale and yard long bean. Thus, besides encouraging consumers to wash vegetables before consuming them, they should also be further informed about the current knowledge of cooking as an effective method to reduce pesticide residues. Awareness of the effects of cooking on reducing the risks associated with the intake of pesticide residues also has the potential to encourage consumers to eat more vegetables. This research report is the seventh article in our series on food safety in Thailand. It is our wish that these studies will stimulate awareness and thoughtfulness regarding this food safety problem, specifically in Thailand and neighboring Asian countries.

## Figures and Tables

**Table 1:** Effects of boiling on the removal of pesticide residues from the Chinese kale samples.

Pesticides	Pesticide Residues Found (ppb)		
	Control (Uncooked)	Boiled	% Removal
Carbamates			
Carbofuran	6.9 ± 1.2	4.5 ± 0.9 **	35
Fenobucarb	2787 ± 527	2208 ± 447 *	21
Indoxacarb	120 ± 25	35 ± 7 ***	71
Organophosphates			
Chlorpyrifos	1222 ± 145	1223 ± 311	0
Diazinon	1840 ± 380	25 ± 12,517 **	32
Profenofos	5235 ± 602	4278 ± 619 *	18
Pyrethroids			
λ-Cyhalothrin	73 ± 10	32 ± 4 ***	55
Cypermethrin	169 ± 23	74 ± 9 ***	56
Deltamethrin	255 ± 47	87 ± 14 ***	66
Fenvalerate	33 ± 4	12 ± 2 ***	62
Flumethrin	201 ± 42	182 ± 46	9
Other			
Alachlor	7.7 ± 1.3	3.2 ± 0.7 ***	59
Flutolanil	0.5 ± 0.1	0.4 ± 0.1	14
Mefenacet	0.8 ± 0.2	0.8 ± 0.1	4
Mepronil	464 ± 60	470 ± 121	−1.3
Metalaxyl	1171 ± 196	567 ± 110 ***	52

The results are given as mean ± standard deviation (SD), *n* = 6 each. * Significantly different from the control (uncooked) group, *p* < 0.05. ** Significantly different from the control (uncooked) group, *p* < 0.01. *** Significantly different from the control (uncooked) group, *p* < 0.001.

**Table 2:** Effects of blanching on the removal of pesticide residues from the Chinese kale samples.

Pesticides	Pesticide Residues Found (ppb)		% Removal
	Control (Uncooked)	Blanched	
Carbamates			
Carbofuran	5.9 ± 1.2	1.8 ± 0.1 **	69
Fenobucarb	1629 ± 336	860 ± 156 **	47
Indoxacarb	97 ± 18	75 ± 8	22
Organophosphates			
Chlorpyrifos	1258 ± 258	1005 ± 198	20
Diazinon	1067 ± 204	1128 ± 205	−5.7
Profenofos	4015 ± 739	4029 ± 987	−0.3
Pyrethroids			
λ-Cyhalothrin	70 ± 11	44 ± 9 **	37
Cypermethrin	228 ± 32	136 ± 23 **	40
Deltamethrin	392 ± 58	215 ± 44 **	45
Fenvalerate	42 ± 6	27 ± 5 **	37
Flumethrin	122 ± 20	78 ± 14 **	36
Other			
Alachlor	6.3 ± 1.2	0.0 ± 0 ***	100
Flutolanil	0.4 ± 0.1	0.4 ± 0.1	0
Mefenacet	0.6 ± 0.1	0.6 ± 0.1	0
Mepronil	192 ± 35	206 ± 33	−7.3
Metalaxyl	953 ± 192	198 ± 33 ***	79

The results are given as mean ± standard deviation (SD), *n* = 6 each. ** Significantly different from the control (uncooked) group, *p* < 0.01. *** Significantly different from the control (uncooked) group, *p* < 0.001.

**Table 3:** Effects of stir-frying on the removal of pesticide residues from the Chinese kale samples.

Pesticides	Pesticide Residues Found (ppb)		% Removal
	Control (Uncooked)	Stir-Fried	
Carbamate			
Carbofuran	10.6 ± 2.0	8.0 ± 1.4 *	25
Fenobucarb	2862 ± 507	2123 ± 367 *	26
Indoxacarb	95 ± 16	62 ± 12 **	35
Organophosphates			
Chlorpyrifos	1524 ± 149	1049 ± 181 **	31
Diazinon	2638 ± 373	1530 ± 311 ***	42
Profenofos	6071 ± 750	3635 ± 728 ***	40
Pyrethroids			
λ-Cyhalothrin	91 ± 16	36 ± 7 ***	60
Cypermethrin	212 ± 29	154 ± 31 **	28
Deltamethrin	342 ± 66	178 ± 32 **	48
Fenvalerate	35 ± 6	25 ± 5 **	30
Flumethrin	168 ± 30	345 ± 80	−105
Other			
Alachlor	9.7 ± 2.0	4.8 ± 1.0 ***	51
Flutolanil	0.6 ± 0.1	0.3 ± 0.1 ***	51
Mefenacet	0.7 ± 0.2	0.5 ± 0.1	32
Mepronil	285 ± 41	254 ± 51	11
Metalaxyl	1379 ± 222	939 ± 19 **	32

The results are given as mean ± standard deviation (SD), *n* = 8 each. * Significantly different from the control (uncooked) group, *p* < 0.05. ** Significantly different from the control (uncooked) group, *p* < 0.01. *** Significantly different from the control (uncooked) group, *p* < 0.001.

**Table 4:** Effects of boiling, blanching and stir-frying on removing pesticide residues in yard long bean.

Pesticides	Pesticide Residues Found (ppb)			
	Control (Uncooked)	Boiled	Blanched	Stir-Fried
Organophosphates				
Chlorpyrifos	4.3 ± 0.7	2.7 ± 0.5 *** (37%) ^a^	3.2 ± 0.6 * (27%)	3.5 ± 0.6 (19%)
Dimethoate	156 ± 32	119 ± 19 (24%)	126 ± 26 (20%)	183 ± 30 (−17%)
Profenofos	1.5 ± 0.1	1.4 ± 0.2 (6%)	1.6 ± 0.3 (−7%)	1.4 ± 0.3 (6%)
Other				
Flumethrin	128 ± 25	47 ± 8 *** (63%)	52 ± 10 *** (59%)	84 ± 10 *** (34%)
Captan	4335 ± 446	0.0 ± 0.0 *** (100%)	851 ± 542 *** (80%)	1603 ± 263 *** (63%)

The results are given as mean ± standard deviation (SD), *n* = 8 each. ^a^ % Removal by each cooking process is given in the parenthesis underneath the mean and SD values. * Significantly different from the control (uncooked) group, *p* < 0.05. *** Significantly different from the control (uncooked) group, *p* < 0.001.

**Table 5:** Pesticides found in Chinese kale and yard long bean, and their lipophilicity as reflected by Log *p* (partition coefficient) values.

Chemical Class	Pesticide	Found In		Log *p* ^#^
		Chinese Kale	Yard Long Bean	
Carbamates	Carbofuran	✓		1.8
	Fenobucarb	✓		2.8
	Indoxacarb	✓		4.7
Organophosphates	Chlorpyrifos	✓	✓	4.7
	Diazinon	✓		3.7
	Dimethoate		✓	0.75
	Profenofos	✓	✓	1.7
Pyrethroids	Cypermethrin	✓		5.6
	Deltamethrin	✓		4.6
	Fenvalerate	✓		5.0
	Flumethrin	✓	✓	7.6
	λ-Cyhalothrin	✓		6.8
Others	Captan		✓	2.5
	Metalaxyl	✓		1.8
	Flutolanil	✓		3.2
	Mepronil	✓		3.7
	Alachlor	✓		3.1
	Mefenacet	✓		3.2

^#^ Log *p* is partition coefficient (octanol/water) of a pesticide which was obtained from the PubChem Compound Database (2016).

## Data Availability

The datasets generated for this study are available on request to the corresponding author.

## Supplementary Materials

The following supporting information can be downloaded at: https://www.mdpi.com/article/10.3390/foods11101463/s1, Standards of eighty-eight pesticides and two metabolites, namely 4,4′-DDD, 4,4′-DDE, 4,4′-DDT, alachlor, aldrin, α-endosulfan, atrazine, azinphos-ethyl, azoxystrobin, γ-BHC, β-endosulfan, bifenthrin, butachlor, captan, carbaryl, carbofuran (and its two metabolites carbofuran-3-hydroxy and carbofuran-3-keto), carbosulfan, chlordan, chlormefos, chlorothalonil, chlorpyrifos, chlorpyrifos-methyl, chlorthiophos, cyfluthrin, λ-cyhalothrin, cypermethrin, deltamethrin, diazinon, dichlorvos, dicofol, dicrotophos, difenoconazole, dimethoate, edrin, EPN, ethion, fenitrothion, fenobucarb, fenoxycarb, fenthion, fenvalerate, fipronil, flumethrin, flutolanil, folpet, heptachlor, hexaconazole, imidacloprid, indoxacarb, isoprocarb, malathion, mefenacet, mepronil, metalaxyl, methamidophos, methidathion, methiocarb, methomyl, mevinphos, monocrotophos, omethoate, oxyfluorfen, paraoxon-methyl, parathion-ethyl, parathion-methyl, phenthoate, phosalone, pirimicarb, pirimiphos-ethyl, pirimiphos-methyl, prochloraz, profenofos, promecarb, propargite, propiconazole, prothiofos, pyraclostrobin, pyridaben, quintozene, quizalofop-p-ethyl, tebuconazole, thiabendazole, permethrin, triazophos and trifloxystrobin were purchased from Dr. Ehrenstorfer (Augsburg, Germany).

## Abbreviations

EU	European Union
FDA	Food and Drug Administration
GC-MS	Gas chromatography-Mass spectrometer
GCB	graphited carbon black
MS	Mass spectrometer
MRL	maximum residue limits
QuEChERS	Quick Easy Cheap Effective Rugged and Safe
