# Supplementary material for: Boiling, Blanching, and Stir-Frying Markedly Reduce Pesticide Residues in Vegetables
Source: Foods. 2022 May 18;11(10):1463. doi: 10.3390/foods11101463 (PMC9141337; doi:10.3390/foods11101463)
Supplement: Supplementary file 1 [file foods-11-01463-s001.zip › foods-1689353-supplementary-done.pdf]

foods

MDPI

Supplementary Materials: Standards of eighty-eight pesticides and two metabolites, namely 4,4′-DDD, 4,4′-DDE, 4,4′-DDT, alachlor, aldrin, α-endosulfan, atrazine, azinphos-ethyl, azoxystrobin, γ-BHC, β-endosulfan, bifenthrin, butachlor, captan, carbaryl, carbofuran (and its two metabolites carbofuran-3-hydroxy and carbofuran-3-keto), carbosulfan, chlordan, chlormefos, chlorothalonil, chlorpyrifos, chlorpyrifos-methyl, chlorthiophos, cyfluthrin, λ-cyhalothrin, cypermethrin, deltamethrin, diazinon, dichlorvos, dicofol, dicrotophos, difenoconazole, dimethoate, edrin, EPN, ethion, fenitrothion, fenobucarb, fenoxycarb, fenthion, fenvalerate, fipronil, flumethrin, flutolanil, folpet, heptachlor, hexaconazole, imidacloprid, indoxacarb, isoprocarb, malathion, mefenacet, mepronil, metalaxyl, methamidophos, methidathion, methiocarb, methomyl, mevinphos, monocrotophos, omethoate, oxyfluorfen, paraoxon-methyl, parathion-ethyl, parathion-methyl, phenthoate, phosalone, pirimicarb, pirimiphos-ethyl, pirimiphos-methyl, prochloraz, profenofos, promecarb, propargite, propiconazole, prothiofos, pyraclostrobin, pyridaben, quintozene, quizalofop-p-ethyl, tebuconazole, thiabendazole, permethrin, triazophos and trifloxystrobin were purchased from Dr. Ehrenstorfer (Augsburg, Germany).
